# Intelligent segmentation and measurement based on U-HRCT to explore the anatomical characteristics of the inner ear in unilateral Meniere’s disease: a retrospective quantitative study

**DOI:** 10.1186/s13244-026-02252-1

**Published:** 2026-03-30

**Authors:** Yan Huang, Xing Zhao, Ruowei Tang, Ning Xu, Heyu Ding, Siwei Yang, Jicheng Wang, Jing Xie, Zhenghan Yang, Li Zhuo, Hongxia Yin, Xiaoguang Li, Zhenchang Wang, Pengfei Zhao

**Affiliations:** 1 Department of Radiology, Beijing Friendship Hospital, Capital Medical University, Beijing, China; 2 School of Information Science and Technology, Beijing University of Technology, Beijing, China; 3 Department of Otolaryngology, Head and Neck Surgery, Beijing Friendship Hospital, Capital Medical University, Beijing, China

**Keywords:** Meniere’s disease, Ultra-high-resolution computed tomography, Magnetic resonance imaging, Endolymphatic hydrops, Intelligent segmentation

## Abstract

**Objectives:**

To investigate inner-ear anatomical features and their association with endolymphatic hydrops (EH) in Meniere’s disease (MD) using ultra-high-resolution CT (U-HRCT) with automated segmentation and measurement.

**Materials and methods:**

We retrospectively analyzed U-HRCT data from 105 unilateral MD patients and 100 normal controls. All patients underwent gadolinium-enhanced MRI for EH grading. The TransUNet network segmented cochlear, vestibular, and semicircular canal structures. Cochlear dimensions were extracted via principal component analysis; vestibular morphology was approximated as an ellipsoid. Anatomical parameters were compared among affected/unaffected MD ears and controls; their correlations with EH severity were assessed. Inner-ear metrics were also compared between MD patients stratified by disease duration (≤ 5 vs. > 5 years).

**Results:**

Compared with controls, both affected and unaffected MD ears showed greater cochlear height and smaller modiolus–lateral semicircular canal angles (all *p* < 0.05), with no difference between MD ears. Cochlear diameters and vestibular dimensions did not differ among groups. Patients with disease duration > 5 years exhibited larger vestibular volume, length B, and AB/BC planar areas (all *p* < 0.05). Vestibular EH severity correlated positively with the modiolus–superior semicircular canal angle (r = 0.243, *p* = 0.031) and negatively with AB–posterior and AB–superior semicircular canal angles (r = −0.251, *p* = 0.026 for both).

**Conclusions:**

MD is associated with distinct spatial alterations in the inner ear, which may disrupt endolymphatic dynamics and may contribute to EH development. Long disease duration is linked to selective vestibular expansion, whereas key angular relationships remain stable across time.

**Critical relevance statement:**

We identified correlations between vestibular-hydrops severity and specific inner-ear angular metrics, suggesting links between anatomical spatial relationships and Meniere’s disease (MD) progression. These structural deviations may underlie the pathophysiological basis of MD, affecting endolymphatic flow dynamics and contributing to endolymphatic hydrops development.

**Key Points:**

- Endolymphatic hydrops (EH) is a hallmark of Meniere’s disease (MD).
- We observed cochlear-height and spatial-positioning alterations in inner-ear structures in MD.
- These findings suggest links between anatomical spatial relationships and MD progression.

**Graphical Abstract:**

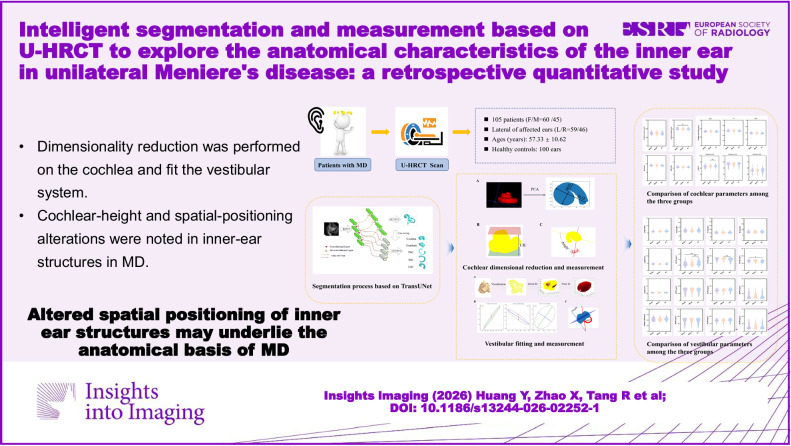

## Background

Meniere’s disease (MD) is a chronic inner-ear disorder characterized by recurrent vertigo, fluctuating hearing loss, tinnitus, and aural fullness [1]. The prevalence of MD is approximately 50–200 cases per 100,000 adults, with the most common age of onset between 40 and 60 years [2]. Endolymphatic hydrops (EH) is widely recognized as the primary pathological feature of MD [3, 4]. In 2007, Nakashima et al [5] first demonstrated EH in patients with MD using three-dimensional fluid-attenuated inversion recovery MRI, enabling direct visualization of EH in affected patients.

Despite these advances, the etiology of MD and pathogenesis of EH remain unclear and are believed to involve multiple factors, including inner-ear anatomical variations, immune abnormalities, genetic predisposition, viral infections, and homeostatic imbalance [6, 7]. Anatomical variations of the inner ear are thought to play significant roles in the development and progression of MD. Previous studies have reported that patients with MD may exhibit more indistinct or narrower vestibular aqueducts [8, 9], a shorter distance between the posterior fossa and the vertical part of the posterior semicircular canal (PSC) [10], and a higher incidence of jugular bulb anomalies [11]. Research on cochlear and vestibular anatomy in MD remains limited, with most evaluations relying on two-dimensional CT measurements [12], which inadequately capture the spatial positioning and complex morphological architecture of the inner ear in MD.

Recent advances in imaging technologies, particularly ultra-high-resolution CT (U-HRCT), have enabled detailed visualization of inner-ear anatomical structures. U-HRCT enables isotropic voxel reconstruction of 0.1 × 0.1 × 0.1 mm with a minimum slice thickness of 0.05 mm, allowing clear visualization of the bony anatomy of the ear [13, 14]. However, traditional manual measurement methods have high subjectivity, low efficiency, and poor reproducibility, making them unsuitable for large-scale clinical studies. Consequently, intelligent segmentation and measurement techniques have emerged as solutions to provide automated and precise analysis of inner-ear anatomical structures [15, 16].

In this study, we utilized U-HRCT images to automatically segment the inner-ear substructures in patients with MD and measured structural characteristics of the cochlea and vestibule. We hypothesized that patients with MD exhibit specific inner-ear anatomical variations that are associated with EH severity. We anticipate that the findings of this study will contribute to a more comprehensive understanding of the complex anatomical factors involved in MD and provide new insights into potential diagnostic strategies.

## Materials and methods

### Patients

This retrospective case-control study analyzed the patients with unilateral MD who underwent inpatient evaluation at Beijing Friendship Hospital between July 2022 and October 2024. The inclusion criteria for MD were as follows: (1) definite MD diagnosis according to the 2015 Bárány Society diagnostic criteria [17]; (2) unilateral involvement only; (3) availability of U-HRCT scans; and (4) gadolinium-enhanced MRI. The exclusion criteria were as follows: (1) poor visualization on CT/MRI, (2) concurrent middle ear pathologies (e.g., otitis media, cholesteatoma), and (3) a history of ear trauma or prior otologic surgery.

HCs were selected from individuals who underwent temporal bone U-HRCT for non-inner-ear-related clinical indications. Specific indications included: (1) evaluation of contralateral conductive hearing loss; (2) workup for unilateral non-pulsatile tinnitus or subjective hearing disturbance where audiological and radiological evaluation of the index ear ruled out inner-ear pathology; and (3) preoperative planning for suspected middle ear or mastoid pathology. All potential HC ears underwent rigorous radiological review by two experienced neuroradiologists to confirm the absence of any inner-ear structural anomaly, otosclerosis, or other relevant temporal bone pathology before inclusion. The study protocol was approved by the Ethics Committee of Beijing Friendship Hospital, affiliated with Capital Medical University (No. 2022-P2-259-02).

### U-HRCT scan

All participants underwent bilateral temporal bone imaging using an ultra-high-resolution CT (U-HRCT) scanner, specifically a cone-beam CT system (LargeV Instrument Corp.). Scans were performed in the supine position using the small-field-of-view (FOV) high-definition mode, with bilateral temporal bones imaged separately. The scanning range extended from the petrous bone arcuate eminence superiorly to the stylomastoid foramen inferiorly, with the following technical parameters: tube voltage, 100 kV; tube current, 3.5 mA; reconstructed voxel size, 0.1 × 0.1 × 0.1 mm; reconstruction FOV: 65 × 65 mm; and total slices, 370.

### Segmentation of inner-ear substructures

**Fig. 1:**
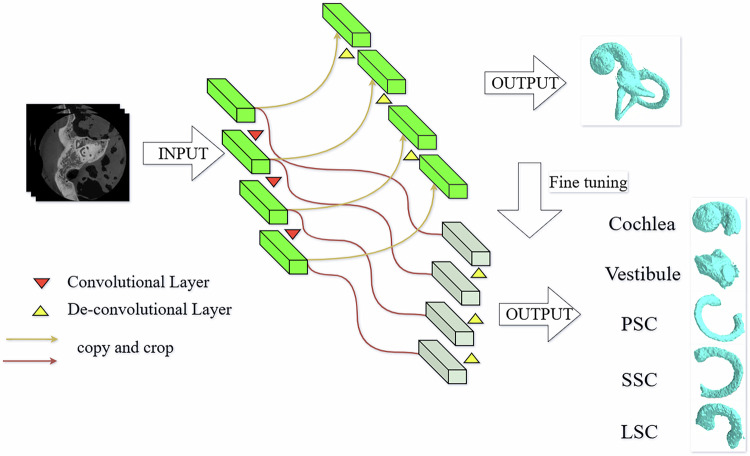
Flowchart of the segmentation process based on TransUNet. PSC, posterior semicircular canal; LSC, lateral semicircular canal; SSC, superior semicircular canal

This study used a TransUNet-based segmentation network architecture using a two-stage segmentation approach. In the first stage, a pretrained TransUNet model was utilized for initial feature extraction and segmentation. This model employs an encoder–decoder structure that learns both global and local features from input three-dimensional U-HRCT images. In the second stage, the inner-ear segmentation model was fine-tuned, with specific adjustments made to the decoder for targeted optimization. Ultimately, five substructures—the cochlea, vestibule, PSC, lateral semicircular canal (LSC), and superior semicircular canal (SSC)—were successfully extracted from the three-dimensional U-HRCT images. Specific segmentation details were obtained from previous studies [18, 19]. Figure 1 illustrates the two-stage segmentation network architecture and workflow based on TransUNet.

### Dimensionality reduction and measurement of cochlear structures

All cochlear voxel coordinates were retrieved as coords1, and their total number was recorded. The voxel count was converted to volume units based on the CT scan resolution (1 voxel = 0.1 mm³). Principal component analysis (PCA) was applied to the cochlear voxel coordinates to reduce dimensionality and achieve spatial calibration.

In the two-dimensional reduced coordinates (coords2), the point with the largest x-coordinate (x₁, y₁) was defined as the center of the round window. The Euclidean distance between each point and the round window center was calculated, and the farthest point (x₂, y₂) was identified. This maximum distance represented the basal long diameter (BLD) of the cochlea. A new coordinate system was then established using the line connecting the two measurement points of the cochlear base along the x-axis. Within this new coordinate system, all points sharing the same x-coordinate were grouped, and the pair of points with the greatest difference in y-coordinate values (x₃, y₃) and (x₄, y₄) was identified. The absolute difference |y₃ − y₄| was defined as the basal short diameter (BSD) of the cochlea. Based on the BLD, the following cochlear duct parameters were derived [20, 21]:

1 $${{\rm{Cochlear}}}\; {{\rm{duct}}}\; {{\rm{length}}}({{\rm{CDL}}})=-4.16\times {{\rm{BLD}}}-4$$

2 $${{\rm{Two}}}-{{\rm{turn}}}\; {{\rm{length}}}(2{{\rm{TL}}})=3.65\times ({{\rm{BLD}}}-1)$$

3 $${{\rm{Base}}}-{{\rm{turn}}}\; {{\rm{length}}}({{\rm{BTL}}})=2.43\times ({{\rm{BLD}}}-1)$$

From the spatially calibrated coordinates (coords3), the points with the smallest and largest absolute Z values were identified. The point with min|Z| (x₃, y₃, z₃) represented the cochlear apex, while the point with max|Z| (x₄, y₄, z₄) represented the cochlear base. The cochlear height (CH) was calculated as CH = max|Z| − min|Z|. The modiolar axis of the cochlea was represented as the vector (xc, yc, zc). The angle (θ) between this vector and each of the three semicircular canals was calculated using the formula:

$${{{\rm{\theta }}}=\cos }^{-1}\frac{{x}_{c}\times x+{y}_{c}\times y+{z}_{c}\times z}{\sqrt{{{x}_{c}}^{2}+{{y}_{c}}^{2}+{{z}_{c}}^{2}}\sqrt{{x}^{2}+{y}^{2}+{z}^{2}}}$$

**Fig. 2:**
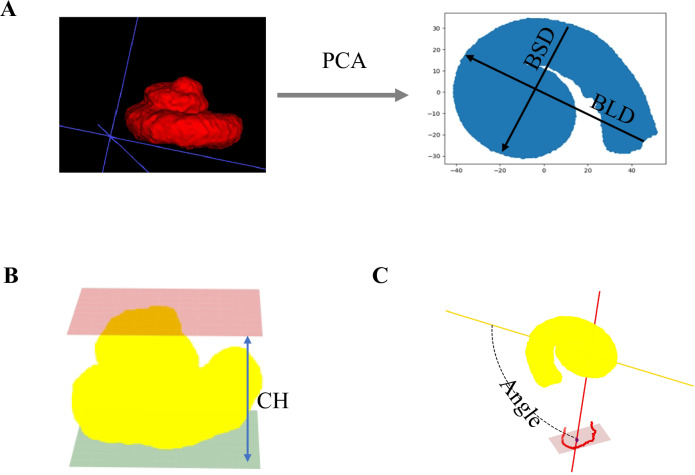
Cochlear dimensional reduction and measurement of related indicators. **A** The cochlea is reduced to a two-dimensional plane using PCA, and the BLD and BSD are measured. **B** The CH is measured as the vertical distance between the voxels with the maximum and minimum z-coordinates. **C** The modiolus and lateral semicircular canal are fitted to planes, and the angle between them is measured. PCA, principal component analysis; BLD, basal long diameter; BSD, basal short diameter; CH, cochlear height

Details of the cochlear measurement parameters are shown in Fig. 2.

### Fitting and measurement of vestibular structures

**Fig. 3:**
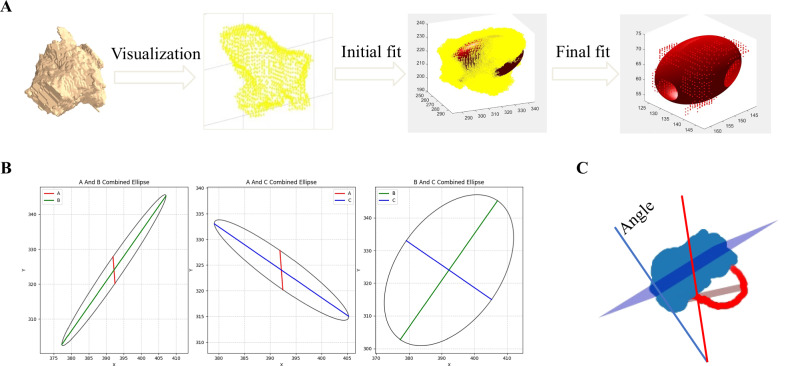
Fitting of vestibular structures and measurement of related parameters. **A** The vestibular structure is voxelized, followed by ellipsoid fitting with iterative optimization. **B** The fitted ellipsoid is equally divided along three orthogonal directions, yielding three cross-sectional planes designated as AB, AC, and BC. **C** Measured the relative angle between the horizontal semicircular canal and the ellipsoidal plane

Given the irregular, yet approximately ellipsoidal morphology of the vestibular system, an ellipsoid model approximation was adopted for algorithmic development (Fig. 3). Ellipsoid fitting of the vestibular voxel point clouds and subsequent statistical analyses were conducted in three main methodological stages.

#### Step 1: Data preprocessing and ellipsoid equation formulation

First, vestibular voxel point sets were extracted from the raw data to represent discrete samples of the ellipsoidal surface, with vestibular volume calculated based on voxel counts. The canonical ellipsoid equation (x/a)^2^ + (y/b)^2^ + (z/c)^2^ = 1 (where *a*, *b*, and *c* denote the semi-principal axis radii) was then transformed into a generalized linear equation. This conversion involved algebraic substitutions introducing new variables (e.g., X = x^2^, Y = y^2^, Z = z^2^) and coefficients (e.g., A = 1/a^2^, B = 1/b^2^, C = 1/c^2^), thereby reformulating the original equation as a linear combination AX + BY + CZ = 1.

#### Step 2: Linear system construction and least-squares fitting

The preprocessed voxel point sets were substituted into the transformed linear equations. Each point contributed an equation to the system, resulting in an overdetermined system in which the number of equations exceeded the number of unknowns. A design matrix was constructed to include the squared coordinates and cross terms of all points, along with a response vector (typically a constant or linear combination). The linear least-squares problem was then solved to determine the coefficients of the ellipsoid equation.

#### Step 3: Metric quantification

From the solved coefficients, the three principal diameters of the ellipsoid were inversely calculated and denoted as A, B, and C. Using these diameters, three planar cross-sectional areas (AB, AC, and BC) were computed. The fitted ellipsoid planes were further analyzed by calculating their angles relative to the three semicircular canals using surface normal vectors.

### MRI scanning and EH evaluation

**Fig. 4:**
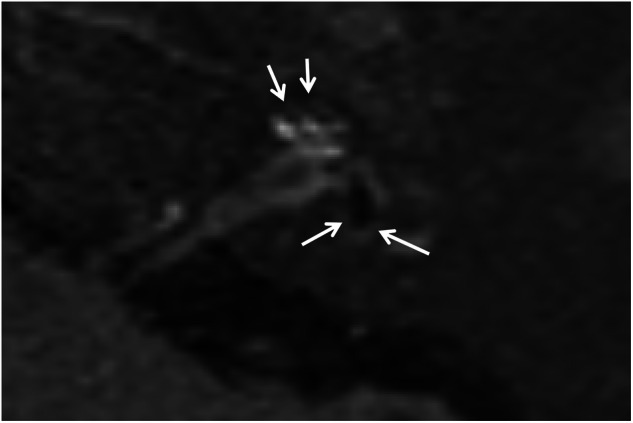
Endolymphatic hydrops in the cochlea and vestibule on gadolinium-enhanced magnetic resonance. Endolymphatic hydrops is observed at grade 2 in the cochlea and grade 3 in the vestibule of the patient (white arrows)

A gadolinium-based contrast agent (gadopentetate dimeglumine; Bayer Pharmaceuticals) was administered intratympanically into both ears at a dose of 0.5 mL per ear (diluted 1:7 with saline), and an MRI was performed 24 h later using a Siemens 3-T MRI scanner equipped with a 64-channel head coil. The imaging parameters were as follows: TE = 187 ms, TR = 6000 ms, TI = 1730 ms, slice thickness = 0.8 mm, FOV = 200 × 200 mm², PAT = 2, and total scan time = 6 min 38 s. The degree of cochlear EH was classified into four grades according to Gurkov et al [22], whereas vestibular EH was categorized into three grades following the criteria of Nakashima et al [23]. Figure 4 presents the gadolinium-enhanced MRI findings of a patient with MD.

### Pure tone audiometry

The test was conducted a few days before the MRI in a standard soundproof chamber with background noise below 25 dB(A). Hearing thresholds for air conduction were measured using an audiometer, and the pure tone average (PTA) for both ears was calculated as the mean of the 500, 1000, 2000, and 4000 Hz thresholds.

### Statistical analysis

Statistical analyses were performed using SPSS version 25.0. Quantitative variables with normal distribution are expressed as mean ± standard deviation. Categorical variables are described as frequencies and percentages (*n* (%)). One-way analysis of variance (ANOVA) was used to compare cochlear and vestibular parameters among three groups: MD–affected ears (MDAE), MD–unaffected ears (MDUE), and HC, followed by post hoc pairwise comparisons. Spearman’s correlation analysis was conducted to evaluate the relationship between EH severity and cochlear or vestibular measurements. To assess the potential influence of disease duration, patients were stratified into two subgroups (duration ≤ 5 years vs. > 5 years), and intergroup comparisons of anatomical parameters were made. Statistical significance was set at *p* < 0.05.

## Results

### Participants

This study included 105 patients with unilateral MD, comprising 60 females and 45 males, aged 30 to 77 years (mean age: 57.33 ± 10.62 years). Among the 105 affected ears, 56.19% were on the left and 43.81% were on the right. Regarding disease duration (defined as the interval from the first reported onset of definitive MD symptoms to the time of imaging), 3.81% of patients had symptoms for less than 1 year, 40.95% had symptoms for 1–5 years, and 55.24% had symptoms for more than 5 years. In terms of treatment, 14.29% of patients received pharmacological therapy, while 85.71% underwent surgical intervention. Detailed clinical characteristics are presented in Table 1. In addition, 100 radiologically confirmed normal ears from the meticulously selected HC cohort (see “Materials and methods” for detailed selection criteria) were included. There were no significant differences in sex or age between the MD patient group and the HC group (*p* > 0.05).

**Table 1:** General demographic and clinical characteristics

Clinical characteristics	Patients (mean ± SD; *n*, %)
Age (years)	57.33 ± 10.62
Sex	
Female	60 (57.14%)
Male	45 (42.86%)
Duration of disease	
< 1 year	4 (3.81%)
1–5 years	43 (40.95%)
≥ 5 years	58 (55.24%)
Laterality of the affected ear	
Left	59 (56.19%)
Right	46 (43.81%)
PTA (dB HL)	
Affected ear	65.69 ± 18.09
Unaffected ear	23.64 ± 14.09
Clinical stage	
Stage I	1 (0.95%)
Stage II	6 (5.71%)
Stage III	51 (48.57%)
Stage IV	47 (44.76%)
Treatment modality	
Pharmacotherapy	15 (14.29%)
Surgical intervention	90 (85.71%)

*PTA* pure tone average

### Comparison of cochlear parameters among groups

**Fig. 5:**
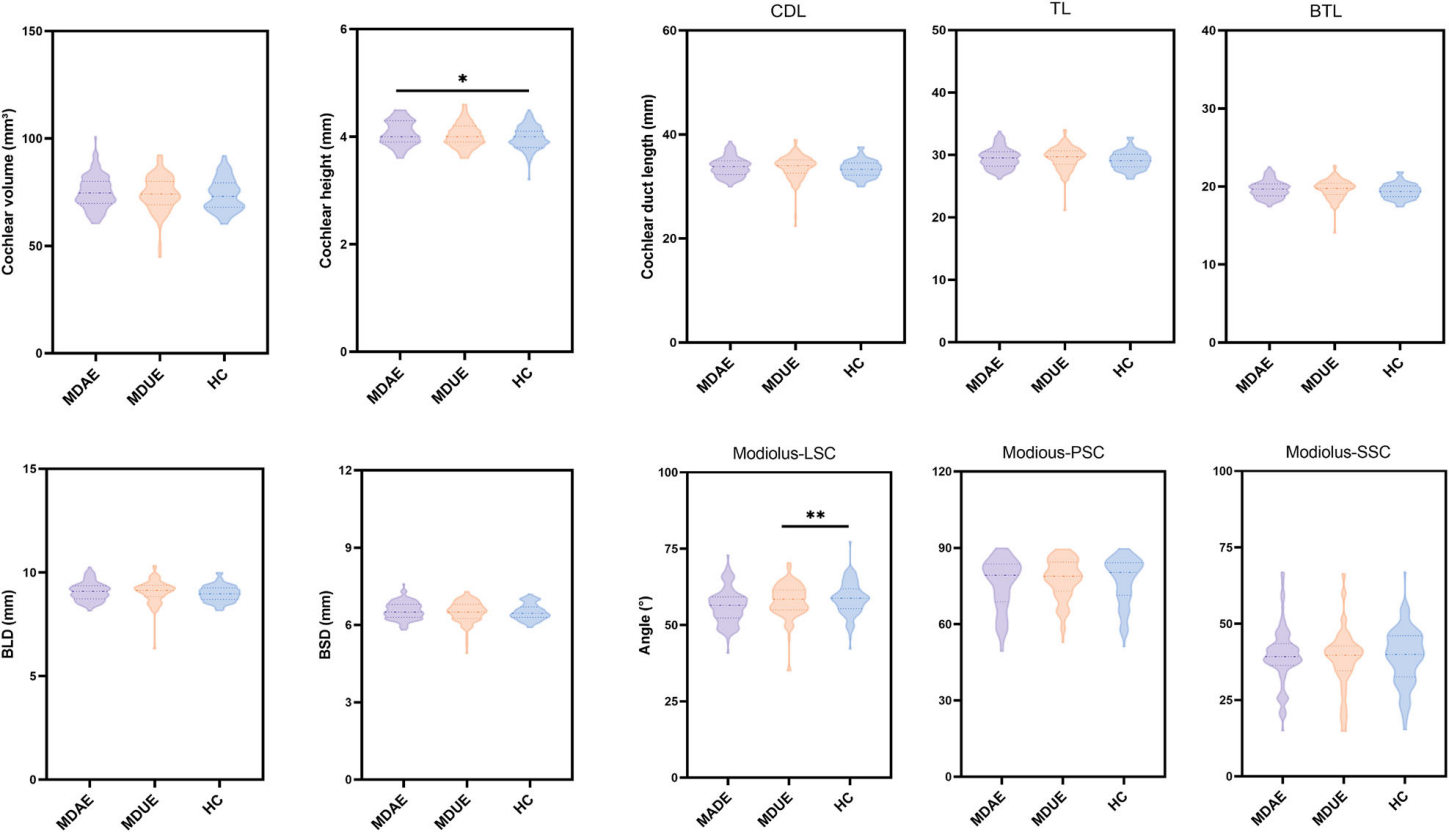
Comparison of cochlear parameters among the three groups. MDAE, Meniere’s disease–affected ears; MDUE, Meniere’s disease–unaffected ears; HC, healthy controls; BLD, basal long diameter; BSD, basal short diameter; PSC, posterior semicircular canal; LSC, lateral semicircular canal; SSC, superior semicircular canal; CDL, cochlear duct length; 2TL, two-turn length; BTL, base-turn length. * *p* < 0.05; ** *p* < 0.01

Figure 5 and Table 2 present the cochlear indicators of the MDAE, MDUE, and HC groups. As shown in Table 2, the ANOVA indicated a significant group difference in CH (F = 3.202, *p* = 0.042). However, post hoc multiple comparison tests confirmed that the only significant difference was between the HC and MDAE groups (*p* = 0.036). For the angle between the modiolus and LSC, ANOVA and post hoc analyses revealed significant differences between the HC and MDUE groups (*p* < 0.01), while no other group differences were observed (*p* > 0.05). Furthermore, no significant differences were found among the three groups for other cochlear-related indicators (*p* > 0.05).

**Table 2:** Comparison of cochlear parameters across the three groups

	MDAE	MDUE	HC	F-value	*p*-value
CH (mm)	4.06 ± 0.22	4.02 ± 0.23	3.98 ± 0.22	3.202	0.042*
Cochlear volume (mm^3^)	75.01 ± 7.76	74.12 ± 8.36	73.82 ± 7.49	0.625	0.536
BLD (mm)	9.08 ± 0.44	9.03 ± 0.65	8.98 ± 0.38	0.999	0.370
BSD (mm)	6.55 ± 0.33	6.50 ± 0.41	6.50 ± 0.30	0.749	0.474
CDL (mm)	33.77 ± 1.86	33.60 ± 2.71	33.35 ± 1.62	1.000	0.369
2TL (mm)	29.49 ± 1.63	29.34 ± 2.37	29.12 ± 1.42	0.999	0.369
BTL (mm)	19.63 ± 1.09	19.53 ± 1.58	19.39 ± 0.94	1.000	0.369
Modiolus–LSC (°)	57.89 ± 5.59	59.07 ± 6.23	56.02 ± 5.81	6.934	0.001**
Modiolus–PSC (°)	73.92 ± 13.47	76.01 ± 11.38	75.73 ± 11.43	0.911	0.403
Modiolus–SSC (°)	41.52 ± 13.48	40.76 ± 13.68	41.28 ± 13.27	0.087	0.916

*MDAE* Meniere’s disease–affected ears, *MDUE* Meniere’s disease–unaffected ears, *HC* healthy controls, *CH* cochlear height, *BLD* basal long diameter, *BSD* basal short diameter, *PSC* posterior semicircular canal, *LSC* lateral semicircular canal, *SSC* superior semicircular canal, *CDL* cochlear duct length, *2TL* two-turn length, *BTL* base-turn length

The bold values indicate statistically significant differences among the three groups (MDAE, MDUE, and HC) for the corresponding cochlear parameters

* *p* < 0.05; ** *p* < 0.01

### Comparison of vestibular parameters among groups

**Fig. 6:**
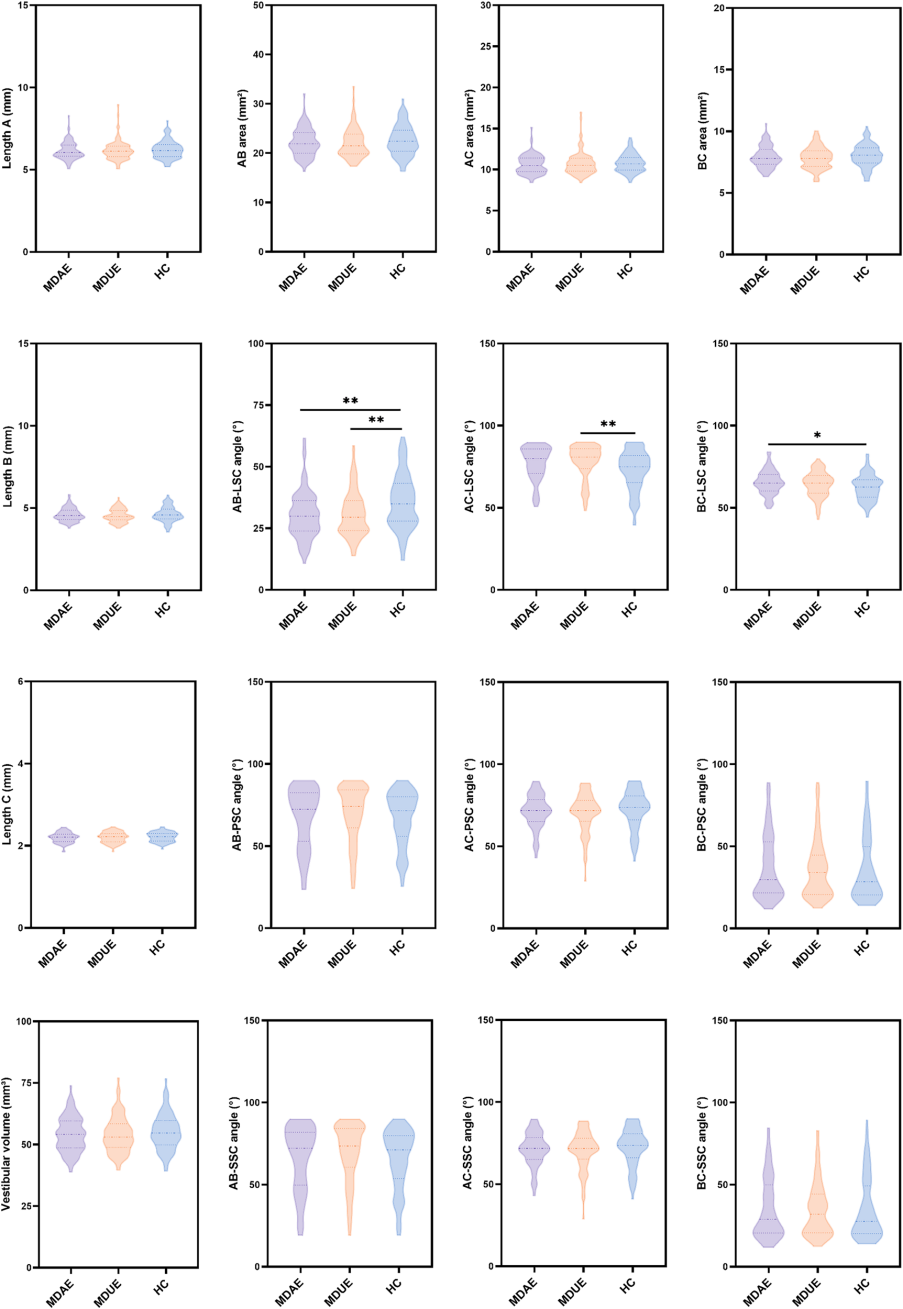
Comparison of vestibular parameters among the three groups. MDAE, Meniere’s disease–affected ears; MDUE, Meniere’s disease–unaffected ears; HC, healthy controls; PSC, posterior semicircular canal; LSC, lateral semicircular canal; SSC, superior semicircular canal. * *p* < 0.05; ** *p* < 0.01

Figure 6 and Table 3 present the differences in vestibular measurement indicators among the three groups. No significant differences were observed among the groups in the diameter, area, or volume of the vestibule. ANOVA indicated a significant difference in the angle between the AB plane and LSC (F = 7.965, *p* < 0.001), and post hoc comparisons showed that the angles in the MDAE and MDUE groups were significantly smaller than those in the HC group (*p* < 0.01), whereas no significant difference was found between the MDAE and MDUE groups (*p* > 0.05). For the angle between the AC plane and the LSC, ANOVA and post hoc comparisons revealed significant differences between the HC and MDUE groups (*p* < 0.01), with no other significant group differences (*p* > 0.05). Regarding the angle between the BC plane and the LSC, ANOVA and post hoc analyses revealed significant differences between the HC and MDAE groups (*p* < 0.05), while no other group differences were observed (*p* > 0.05). No significant differences were detected in the angles between the vestibular plane and either the PSC or SSC (*p* > 0.05).

**Table 3:** Comparison of vestibular-related metrics among the three groups

Parameter	MDAE	MDUE	HC	F-value	*p*-value
Vestibular volume (mm^3^)	54.12 ± 7.09	54.03 ± 7.20	55.11 ± 7.20	0.701	0.497
Length A (mm)	6.14 ± 0.52	6.20 ± 0.68	6.21 ± 0.53	0.432	0.650
Length B (mm)	4.59 ± 0.40	4.54 ± 0.39	4.61 ± 0.46	0.762	0.468
Length C (mm)	2.19 ± 0.12	2.19 ± 0.12	2.21 ± 0.11	0.488	0.614
AB area (mm^2^)	22.17 ± 2.78	22.17 ± 3.38	22.55 ± 3.09	0.514	0.598
BC area (mm^2^)	7.93 ± 0.87	7.86 ± 0.86	8.02 ± 0.91	0.905	0.405
AC area (mm^2^)	10.60 ± 1.13	10.71 ± 1.37	10.79 ± 1.10	0.643	0.526
AB–LSC (°)	31.38 ± 11.91	30.90 ± 9.10	36.60 ± 12.62	7.965	0.000***
AC–LSC (°)	75.60 ± 13.46	77.55 ± 11.00	71.51 ± 14.15	5.801	0.003**
BC–LSC (°)	65.36 ± 7.25	64.28 ± 7.53	62.37 ± 7.69	4.16	0.016*
AB–PSC (°)	64.99 ± 20.85	67.03 ± 20.62	64.65 ± 19.16	0.419	0.658
AC–PSC (°)	71.34 ± 10.11	70.51 ± 11.46	72.46 ± 11.38	0.804	0.448
BC–PSC (°)	36.17 ± 19.61	35.40 ± 17.76	35.76 ± 19.98	0.042	0.959
AB–SSC (°)	64.99 ± 20.85	67.03 ± 20.62	64.65 ± 19.16	0.419	0.658
AC–SSC (°)	71.34 ± 10.11	70.51 ± 11.46	72.46 ± 11.38	0.804	0.448
BC–SSC (°)	36.17 ± 19.61	35.40 ± 17.76	35.76 ± 19.98	0.042	0.959

*MDAE* Meniere’s disease–affected ears, *MDUE* Meniere’s disease–unaffected ears, *HC* healthy controls, *PSC* posterior semicircular canal, *LSC* lateral semicircular canal, *SSC* superior semicircular canal

The bold values indicate statistically significant differences among the three groups (MDAE, MDUE, and HC) for the corresponding vestibular parameters

* *p* < 0.05; ** *p* < 0.01; *** *p* < 0.001

### Correlation between the EH severity and inner-ear parameters

EH was detected in the cochlea of 92.4% of affected ears and 85.7% in the vestibule (Supplementary Table 1). Cochlear EH was predominantly grade 1 (40.00%) or grade 2 (36.19%), with grade 3 observed in 16.19% of ears. Vestibular EH was most frequently grade 1 (47.62%), while 14.29% of ears showed no detectable vestibular EH. Correlation analysis revealed no significant correlations between the degree of cochlear EH and any inner-ear parameters on the affected side in patients with MD (*p* > 0.05). However, the degree of vestibular EH showed a positive correlation with the modiolus–SSC angle (r = 0.243, *p* = 0.031) and negative correlations with both the angle between the AB plane and PSC (r = −0.251, *p* = 0.026) and the angle between the AB plane and SSC (r = −0.251, *p* = 0.026). The positive correlations of hydrops are presented in Table 4.

**Table 4:** Correlations between anatomical parameters and the degree of endolymphatic hydrops

	Degree of cochlear endolymphatic hydrops		Degree of vestibular endolymphatic hydrops	
	Spearman r	*p*-value	Spearman r	*p*-value
CH	0.163	0.143	0.173	0.119
Cochlear volume	0.118	0.290	0.049	0.660
BLD	0.092	0.413	−0.072	0.518
BSD	0.062	0.581	−0.016	0.889
CDL	0.093	0.407	−0.070	0.531
2TL	0.093	0.408	−0.071	0.526
BTL	0.092	0.410	−0.072	0.518
Modiolus–LSC	0.072	0.520	−0.144	0.196
Modiolus–PSC	0.032	0.776	−0.007	0.952
Modiolus–SSC	0.072	0.520	0.243	0.031*
Vestibular volume	0.025	0.824	−0.107	0.339
Length A	0.172	0.121	0.104	0.353
Length B	0.011	0.924	0.154	0.167
Length C	−0.035	0.757	−0.004	0.973
AB area	0.086	0.441	0.172	0.122
BC area	−0.033	0.771	0.099	0.378
AC area	0.095	0.396	0.075	0.503
AB–LSC	0.014	0.902	0.112	0.318
AC–LSC	−0.068	0.544	−0.184	0.098
BC–LSC	−0.040	0.720	0.004	0.972
AB–PSC	–0.068	0.544	−0.251	0.026*
AC–PSC	0.095	0.397	0.198	0.074
BC–PSC	0.047	0.678	0.048	0.671
AB–SSC	–0.040	0.720	−0.251	0.026*
AC–SSC	0.095	0.397	0.198	0.074
BC–SSC	0.047	0.678	0.048	0.671

*CH* cochlear height, *BLD* basal long diameter, *BSD* basal short diameter, *BTL* base-turn length, *CDL* cochlear duct length, *LSC* lateral semicircular canal, *PSC* posterior semicircular canal, *SSC* superior semicircular canal, *2TL* two-turn length

The bold values indicate statistically significant correlations

* *p* < 0.05

### Influence of disease duration on inner-ear parameters

To evaluate whether disease chronicity influences inner-ear anatomy, patients were categorized into short-to-intermediate (≤ 5 years, *n* = 47) and long-duration (> 5 years, *n* = 58) groups. Anatomical parameters on the affected side in patients with MD were compared between these groups using independent samples *t*-tests, as detailed in Table 5. No significant differences were observed in cochlear parameters in relation to disease duration (all *p* > 0.05). Analysis of the vestibular structures revealed a more differentiated outcome. First, regarding spatial orientation, all angular relationships between the vestibular planes (AB, AC, BC) and the semicircular canals (LSC, PSC, SSC) were comparable between groups (all *p* > 0.05). Second, for morphometric (size-related) measures, the long-duration group exhibited slightly larger values in several parameters: vestibular volume (t = 2.002, *p* = 0.048), length B (t = 2.582, *p* = 0.012), AB area (t = 2.114, *p* = 0.037), and BC area (t = 2.722, *p* = 0.009). Length A, length C, and AC area did not differ significantly (*p* > 0.05).

**Table 5:** Comparison of inner-ear anatomical parameters by disease duration

	Short-to-intermediate duration group (≤ 5 years)	Long-duration group (> 5 years)	t-value	*p*-value
CH (mm)	3.98 ± 0.19	4.05 ± 0.24	−1.509	0.134
Cochlear volume (mm^3^)	73.39 ± 7.54	76.32 ± 7.76	−1.951	0.054
BLD (mm)	9.03 ± 0.47	9.12 ± 0.43	−1.022	0.310
BSD (mm)	6.50 ± 0.35	6.60 ± 0.33	−1.439	0.153
CDL (mm)	33.57 ± 1.97	33.95 ± 1.78	−1.024	0.308
2TL (mm)	29.31 ± 1.7	29.64 ± 1.57	−1.024	0.309
BTL (mm)	19.51 ± 1.15	19.74 ± 1.04	−1.026	0.307
Modiolus–LSC (°)	57.93 ± 4.76	57.86 ± 6.22	0.070	0.944
Modiolus–PSC (°)	76.39 ± 10.53	71.93 ± 15.25	1.764	0.081
Modiolus–SSC (°)	40.57 ± 13.91	42.30 ± 13.20	−0.645	0.520
Vestibular volume (mm^3^)	52.62 ± 6.80	55.35 ± 7.14	−2.002	0.048*
Length A (mm)	6.11 ± 0.57	6.17 ± 0.49	−0.539	0.591
Length B (mm)	4.49 ± 0.37	4.68 ± 0.41	−2.582	0.011*
Length C (mm)	2.18 ± 0.10	2.21 ± 0.13	−1.247	0.215
AB area (mm^2^)	21.55 ± 2.7	22.69 ± 2.72	−2.114	0.037*
BC area (mm^2^)	7.69 ± 0.76	8.13 ± 0.91	−2.722	0.008**
AC area (mm^2^)	10.48 ± 1.16	10.71 ± 1.11	−1.062	0.291
AB–LSC (°)	30.45 ± 9.81	32.15 ± 13.41	−0.751	0.454
AC–LSC (°)	77.03 ± 10.12	74.45 ± 15.66	1.019	0.311
BC–LSC (°)	64.73 ± 7.47	65.88 ± 7.10	−0.803	0.424
AB–PSC (°)	67.63 ± 21.38	62.85 ± 20.35	1.163	0.248
AC–PSC (°)	71.46 ± 10.51	71.26 ± 9.88	0.098	0.923
BC–PSC (°)	34.50 ± 19.28	37.53 ± 19.95	−0.788	0.433
AB–SSC (°)	67.63 ± 21.38	62.85 ± 20.35	1.163	0.248
AC–SSC (°)	71.46 ± 10.51	71.26 ± 9.88	0.098	0.923
BC–SSC (°)	34.50 ± 19.28	37.53 ± 19.95	−0.788	0.433

*CH* cochlear height, *BLD* basal long diameter, *BSD* basal short diameter, *BTL* base-turn length, *CDL* cochlear duct length, *LSC* lateral semicircular canal, *PSC* posterior semicircular canal, *SSC* superior semicircular canal, *2TL* two-turn length

The bold values indicate statistically significant differences between the short-to-intermediate duration group (≤ 5 years) and the long-duration group (5 years) for the corresponding parameters

* *p* < 0.05; ** *p* < 0.01

## Discussion

The TransUNet architecture integrates convolutional neural networks with transformer models, offering robust extraction capabilities that are particularly suitable for complex medical imaging data [24]. In the first stage encoder, the model progressively extracted high-dimensional features via multilayer convolutional operations, effectively preserving crucial spatial and contextual information [25], while the decoder used up-sampling and skip connections to gradually reconstruct features to their original image resolution. During the second fine-tuning stage, we modified the decoder’s convolutional layers and activation functions to better align with inner-ear anatomical characteristics and applied data augmentation techniques, including random rotation, translation, and scaling, to expand the training dataset and enhance the model’s generalizability across variations [26]. Additionally, we incorporated an attention mechanism to strengthen the model’s focus on key anatomical regions, enabling better detection of subtle structures and improving segmentation precision and reliability [27].

The snail shell-like morphology of the cochlea and its inherent spatial orientation make parameter measurements challenging. PCA, a classical multivariate statistical technique, effectively reduces high-dimensional data into lower dimensions while preserving essential information [28, 29]. It estimates morphological features by calculating eigenvectors and eigenvalues from the covariance matrix of cochlear spatial coordinates. The three principal component vectors derived from PCA further facilitated an affine transformation model to geometrically correct cochlear CT images. Spatial calibration is essential for aligning the cochlea so that its duct spirals along the Z-axis (with the modiolus approximately perpendicular to the XOY plane), a configuration referred to as the cochlear coordinate system [30]. This calibration process standardizes the spatial orientation of the cochlea, providing a consistent basis for subsequent structural parameter measurements and morphological analyses, thereby greatly enhancing accuracy and reliability.

The vestibular anatomy displays complex and irregular morphological characteristics, marked by multiple curvatures and undulations. This structural complexity presents significant challenges for direct measurement and analysis. Previous studies using two-dimensional CT imaging techniques could only measure the planar parameters of the vestibular system and failed to capture its complete three-dimensional structure [31, 32]. Alternative approaches employing third-party software for three-dimensional reconstruction and measurement, while capable of generating 3D inner-ear models, often involve cumbersome procedures that are highly susceptible to human interference [33]. This study addresses these limitations by modeling the vestibular system as an ellipsoid, thereby simplifying it into a mathematically defined geometric form that retains essential morphological features relevant to overall function.

Although anatomical variations may contribute to the pathogenesis of MD, its exact etiology remains unclear. Previous studies have primarily examined abnormalities of the vestibular aqueduct and endolymphatic sac in MD [7]; however, research specifically addressing inner-ear anatomical variations, particularly in the cochlea and vestibule, remains limited. By combining U-HRCT with automated segmentation and measurement techniques, we systematically investigated the anatomical variations of the inner ear in patients with MD. Our results demonstrated that CH was significantly greater in patients with MD compared to HCs. We hypothesize that this increase may reflect chronic EH-related dilation of the cochlear duct, leading to compression of the perilymphatic space and secondary osseous expansion. This observation aligns with the findings of Krombach et al [12], who also reported elevated CH in the left ear of patients with MD relative to controls. Aside from CH, however, our study did not reveal significant differences in other cochlear structural parameters. Interestingly, while Sugihara et al [34] reported a longer vestibular length and smaller vestibular width in patients with MD using MRI, our measurements did not demonstrate substantial alterations in the vestibular morphology. We propose that this discrepancy may stem from the inherent morphological irregularity of the vestibule, where ellipsoid fitting approaches may overlook some anatomical details, leading to potential deviations between the measured values and the true morphology.

Our study revealed significant alterations in spatial positioning, particularly in the angular relationships between the cochlear modiolus and the horizontal semicircular canal, as well as the relative angles between the vestibular planes and the semicircular canals. Our previous study also identified spatial abnormalities in semicircular canal angles in MD [35]. These positional alterations may influence the circulation and flow dynamics of endolymph within the labyrinthine system and could contribute to the development of hydrops. Existing evidence suggests that EH typically begins at the cochlear apex and subsequently progresses to the vestibule [36]. Previous studies have reported an approximately 80% prevalence of EH in patients with MD [37], with hydrops severity closely correlating with clinical manifestations; greater hydrops are associated with more pronounced hearing loss [38] and longer vertigo duration [39]. Our findings further demonstrate a significant correlation between the severity of vestibular hydrops and vestibular spatial orientation angles. Anatomically, the vestibule directly connects to the vestibular aqueduct and plays a crucial role in endolymph drainage. We propose that altered vestibular spatial positioning may disrupt endolymphatic microdynamics, thereby impairing both circulation and absorption processes.

Stratified analyses based on disease duration revealed that the bony architecture of the cochlea remains largely stable during disease progression, whereas vestibular morphometric parameters exhibit pronounced duration-dependent changes. These changes may reflect adaptive vestibular remodeling in response to chronic endolymphatic pressure. Although endolymph does not exhibit prominent bulk flow comparable to that of blood, biomechanical forces may still be transmitted through hydrostatic mechanisms within the endolymphatic compartment [40]. While the anatomical framework of the inner ear is primarily established during embryogenesis, accumulating evidence from developmental biology indicates that the maintenance and fine-tuning of inner-ear structures are subject to continuous regulation by dynamic extracellular matrix remodeling, local fluid homeostasis, and epithelial–mesenchymal interactions [41]. In addition, MD has been associated with elevated levels of various inflammatory cytokines [42], and a persistently low-grade inflammatory microenvironment may further influence local bony and membranous structures over time.

### Limitations

This study had some limitations. First, the sample size was relatively small; therefore, future studies should include larger cohorts to validate these findings. Second, the measurement of certain parameters requires further refinement. For instance, the CDL was primarily estimated based on BLD, and more precise direct measurements should be performed in future research. Finally, although multiple methods exist for evaluating EH, this study utilized only a semi-quantitative approach. Future investigations should aim to quantify EH volume and explore its correlation with inner-ear parameters.

## Conclusions

This study used U-HRCT combined with automated segmentation and measurement techniques to systematically investigate anatomical variations of the inner ear in unilateral MD. Our findings revealed that patients with MD exhibited significant alterations in CH and spatial positioning of the inner-ear structures compared with HCs. These structural deviations may underlie the pathophysiological basis of MD by affecting endolymphatic flow dynamics and contributing to the development of hydrops.

## Supplementary information

ELECTRONIC SUPPLEMENTARY MATERIAL

## Data Availability

The data that support the findings of this study are available from the corresponding author upon reasonable request.

## Abbreviations

2TL: Two-turn length
ANOVA: Analysis of variance
BLD: Basal long diameter
BSD: Basal short diameter
BTL: Base-turn length
CDL: Cochlear duct length
CH: Cochlear height
CT: Computed tomography
EH: Endolymphatic hydrops
FOV: Field of view
HC: Healthy control(s)
HRCT: High-resolution computed tomography
LSC: Lateral semicircular canal
MD: Meniere’s disease
MDAE: Meniere’s disease–affected ear
MDUE: Meniere’s disease–unaffected ear
MRI: Magnetic resonance imaging
PCA: Principal component analysis
PSC: Posterior semicircular canal
SSC: Superior semicircular canal
U-HRCT: Ultra-high-resolution computed tomography
