# Supplementary material for: Intelligent segmentation and measurement based on U-HRCT to explore the anatomical characteristics of the inner ear in unilateral Meniere’s disease: a retrospective quantitative study
Source: Insights Imaging. 2026 Mar 30;17:83. doi: 10.1186/s13244-026-02252-1 (PMC13035983; doi:10.1186/s13244-026-02252-1)
Supplement: Supplementary file 1 — ELECTRONIC SUPPLEMENTARY MATERIAL [file 13244_2026_2252_MOESM1_ESM.pdf]

Intelligent segmentation and measurement based on U-HRCT to explore the anatomical characteristics of the inner ear in unilateral Meniere's disease: a retrospective quantitative study

ELECTRONIC SUPPLEMENTARY MATERIAL

Supplementary Table 1. Distribution of endolymphatic hydrops grades in patients with unilateral Meniere's disease (n=105 affected ears)

Location of EH	EH Grade	Number of Ears (n)	Percentage (%)
Cochlear EH	Grade 0	8	7.62
	Grade 1	42	40.00
	Grade 2	38	36.19
	Grade 3	17	16.19
Vestibular EH	Grade 0	15	14.29
	Grade 1	50	47.62
	Grade 2	40	38.10

EH, endolymphatic hydrops
